# Abnormal resting-state functional connectivity of the insula in medication-free patients with obsessive-compulsive disorder

**DOI:** 10.1186/s12888-022-04341-z

**Published:** 2022-11-29

**Authors:** Zilin Zhou, Bin Li, Jiaxin Jiang, Hailong Li, Lingxiao Cao, Suming Zhang, Yingxue Gao, Lianqing Zhang, Changjian Qiu, Xiaoqi Huang, Qiyong Gong

**Affiliations:** 1 Huaxi MR Research Center (HMRRC), Functional and Molecular Imaging Key Laboratory of Sichuan Province, Department of Radiology, West China Hospital of Sichuan University, No.37 Guo Xue Xiang, 610041 Chengdu, China; 2 Department of Psychiatry, West China Hospital of Sichuan University, Chengdu, China; 3 Mental Health Center and Psychiatric Laboratory, West China Hospital of Sichuan University, 610041 Chengdu, China; 4 Psychoradiology Research Unit of the Chinese Academy of Medical Science (2018RU011), West China Hospital of Sichuan University, Chengdu, China; 5 Frontiers Science Center for Disease-related Molecular Network, West China Hospital of Sichuan University, Chengdu, China

**Keywords:** Obsessive-compulsive disorder, Insula, Depression, Functional magnetic resonance imaging, Resting-state functional connectivity

## Abstract

**Background:**

The function of the insula has been increasingly mentioned in neurocircuitry models of obsessive-compulsive disorder (OCD) for its role in affective processing and regulating anxiety and its wide interactions with the classic cortico-striato-thalamo-cortical circuit. However, the insular resting-state functional connectivity patterns in OCD remain unclear. Therefore, we aimed to investigate characteristic intrinsic connectivity alterations of the insula in OCD and their associations with clinical features.

**Methods:**

We obtained resting-state functional magnetic resonance imaging data from 85 drug-free OCD patients and 85 age- and sex-matched healthy controls (HCs). We performed a general linear model to compare the whole-brain intrinsic functional connectivity maps of the bilateral insula between the OCD and HC groups. In addition, we further explored the relationship between the intrinsic functional connectivity alterations of the insula and clinical features using Pearson or Spearman correlation analysis.

**Results:**

Compared with HCs, patients with OCD exhibited increased intrinsic connectivity between the bilateral insula and bilateral precuneus gyrus extending to the inferior parietal lobule and supplementary motor area. Decreased intrinsic connectivity was only found between the right insula and bilateral lingual gyrus in OCD patients relative to HC subjects, which was negatively correlated with the severity of depression symptoms in the OCD group.

**Conclusion:**

In the current study, we identified impaired insular intrinsic connectivity in OCD patients and the dysconnectivity of the right insula and bilateral lingual gyrus associated with the depressive severity of OCD patients. These findings provide neuroimaging evidence for the involvement of the insula in OCD and suggest its potential role in the depressive symptoms of OCD.

**Supplementary Information:**

The online version contains supplementary material available at 10.1186/s12888-022-04341-z.

## Introduction

Obsessive-compulsive disorder (OCD) is a severe and disabling mental disorder characterized by the obsession of recurrent, unwanted and intrusive thoughts, images or urges (obsessions) and excessively repetitive ritualistic behaviors or mental acts that individuals feel compelled to perform in response to obsessions according to rigid rules or to achieve a sense of completeness (compulsions) [1, 2]. The intolerance of uncertainty has been recognized as a central psychological mechanism of OCD, especially related to checking and repeating compulsions [3]. It affects 2.4% of the general population in China and leads to a major health-economic burden for affected individuals, families and society as a whole [4, 5].

In addition to the classical frontal-striatal circuit, limbic or affective processing regions such as the amygdala, insula, and hippocampus and their functional network with the classical circuit have been added to the neuroimaging model of OCD [6–8]. Compared to other emotion-related brain regions, neuroimaging studies of the insula in patients with OCD are lacking even though the insula may be closely associated with aversive or uncomfortable sensations [9], excessive risk aversion [10] and intolerance of uncertainty [11, 12] in OCD patients. Previous studies have pointed out that the impaired affective processing and disrupted emotional regulation of OCD is related to dysfunction of the insula, which has been confirmed by the overactivation of the insula during affective tasks or emotional provocation paradigms in OCD patients compared to healthy controls [13, 14]. Another meta-analysis of fMRI studies from patients with OCD showed that this kind of overactivation of the insula during emotional processing was more pronounced in OCD patients with greater anxiety or with mood comorbidities [15]. Furthermore, in a recent study, Fridgeirsson et al. [16] explored the functional network changes in the brain after deep brain stimulation (DBS) in patients with OCD, and they found that the improvement in mood and anxiety symptoms of OCD following DBS was associated with reduced amygdala-insular functional connectivity, which for the first time elucidated the role of the insula in the mechanisms of OCD from an interventional perspective.

In terms of neuroimaging studies, previous studies have revealed characteristic increased alterations in the structure and local function of the insula in patients with OCD [17, 18]. However, relatively few studies have specifically focused on the whole-brain intrinsic functional connectivity of the insula in OCD patients. Resting-state functional connectivity analysis is a fundamental method for delineating the temporal correlation of spontaneous blood oxygenation level-dependent (BOLD) signals among spatially distributed brain regions during the resting state and has been widely used in numerous mental or psychiatric disorders due to its relatively reliable and reproducible nature [19]. Given that our primary aim was to investigate the specific intrinsic functional connectivity patterns of the insula in OCD according to the vital role of the insula in the pathophysiology of this mental disorder, we decided to perform the seed-based resting-state functional connectivity method in the present research, which is a well-targeted method to test the whole-brain intrinsic connectivity of a specific a priori region [20]. In recent years, several studies involving the intrinsic functional connectivity of the insula in OCD patients yielded inconsistent results [21–25]. However, there were some drawbacks in the previous studies, such as relatively small sample sizes and more or less subject to confounding effects of medication or comorbidity.

Therefore, the objective of the current study was to discern the altered whole-brain intrinsic connectivity patterns of the insula by recruiting a relatively large sample of drug-naïve patients with no comorbidities to exclude the confounding effects of medication and comorbidities. Based on the previous functional neuroimaging findings for the insula in patients with OCD, we hypothesized that the intrinsic functional connectivity patterns of the insula would be elevated in OCD patients relative to healthy controls, and there would be a correlation between the abnormal neuroimaging features and the clinical information, especially the mood-related indicators.

## Methods

### Participants

We recruited 85 unmedicated and comorbid-free OCD patients from the Mental Health Center, West China Hospital, Sichuan University. Two experienced psychiatrists diagnosed the OCD patients on the basis of the Structured Clinical Interview (SCID-I) for the Diagnostic and Statistical Manual of Axis I Mental Disorders, fourth edition (DSM-IV). The inclusion criteria were as follows: [1] age between 18 and 60 years; [2] meeting the DSM-IV criteria for OCD; [3] right-handed by the determination of Edinburgh Handedness Inventory; and [4] medication-naïve or had a washout period of at least 4 weeks from any treatment before the imaging data were acquired. The exclusion criteria were as follows: [1] the existence of any other DSM-IV Axis I diagnosis or neurological diseases; [2] any history of cardiovascular diseases, metabolic disorders, or other major physical illness; [3] substance abuse or dependence; [4] pregnancy; and [5] any contraindications to MRI scanning.

Of these 85 OCD patients, 71 patients were medication-naïve. The other 14 patients had received medication for the treatment of OCD (4 were on clomipramine hydrochloride; 3 were on paroxetine hydrochloride; 3 were on fluoxetine hydrochloride; 3 were on sertraline; and 1 was on three types of drugs including clomipramine hydrochloride, paroxetine hydrochloride and quetiapine fumarate), and all of them were medication free for at least 4 weeks before the MR scanning. The Yale-Brown Obsession-Compulsive Scale (Y-BOCS) [26] was used to assess the severity of OCD symptoms. Depression and anxiety symptoms were measured by the 17-item Hamilton Depression Rating Scale (HAMD) [27] and 14-item Hamilton Anxiety Rating Scale (HAMA) [28], respectively.

In addition, we enrolled 85 age- and sex-matched healthy controls (HCs) from the same sociodemographic circumstances via poster advertisements and examined them with the SCID nonpatient edition. The HC subjects and their first-degree relatives were free of any history or present neurological disorder or mental disorders.

This study was approved by the Ethics Committee of West China Hospital, Sichuan University and the study is done in the accordance with the Declaration of Helsinki. Each participant provided written informed consent to accomplish this study after a complete description of the protocol.

### MRI data acquisition

All participants were scanned using a 3.0-Tesla GE Signa EXCITE scanner equipped with an 8-channel phased-array head coil. Each subject was positioned comfortably in the coil fitted with soft earplugs and foam pads and was instructed to keep his or her eyes closed, remain motionless and do not think of any specific topic.

For each individual, we acquired whole-brain resting-state functional magnetic resonance imaging (rs-fMRI) data using a gradient-echo echo-planar imaging (GRE-EPI) sequence with the following parameters [29]: 30 axial slices and volumes in each run = 200, slice thickness = 5.0 mm with no slice gap, repetition time (TR) = 2000 ms, echo time (TE) = 30 ms, flip angle = 90°, the phase encoding direction was anterior to posterior, matrix size = 64 × 64, voxel size = 3.75 × 3.75 × 5 mm^3^ and field of view (FOV) = 240 × 240 mm^2^. The rs-fMRI scans in the current study did not use field maps. The total acquisition time of the GRE-EPI images was approximately 6.67 min (400 s). Additionally, high-resolution T1-weighted 3D anatomical images were obtained with the following parameters: contiguous coronal slices = 156; slice thickness = 1.0 mm; TR = 8.5 ms; TE = 3.4 ms; flip angle = 12°; matrix size = 256 × 256, voxel size = 0.94 × 0.94 × 1 mm^3^ and field of view (FOV) = 240 × 240 mm^2^.

### Imaging preprocessing

The rs-fMRI data preprocessing was performed by the Data Processing and Analysis for Brain Imaging toolkit (DPABI, version 6.0, http://rfmri.org/dpabi) [30]. For each participant, the first 10 time points were discarded in consideration of signal equilibrium and adaptation to the scanning environment. The remaining images were corrected for acquisition time intervals between slices and for head motion between volumes. To control for the head motion, we performed the motion correction strategies using the mean framewise displacement (FD) approach proposed by previous studies [31, 32], which is a higher-level Friston-24 parameter model [33], including 6 head motion parameters, the previous time point of 6 head motion parameters, and the 12 corresponding squared items. The mean FD values were calculated from the translational and rotational scan-to-scan displacements using three translational parameters and three rotational parameters obtained from realignment steps for each subject. Participants were only included when their rs-fMRI images met the criteria of < 1.5 mm of spatial movement and < 1.5 degrees of rotation in any direction and a mean FD value < 0.2 mm. After head movement correction and quality control, no participant was excluded in either the OCD group or the HC group. Then, these images were spatially normalized to the standard Montreal Neurological Institute (MNI) space, and each voxel was resampled to 3 × 3 × 3 mm^3^ using unified segmentation of individual T1 images[34]. The processed images were smoothed with a full width at half maximum (FWHM) Gaussian kernel of 6 mm. Additionally, we regressed out the head motion parameters, white matter and cerebrospinal fluid (CSF) signals to reduce the effects of nonneuronal BOLD fluctuations. Finally, temporal bandpass filtering (0.01–0.08 Hz) was utilized to decrease the impact of high-frequency physiological noise and very low-frequency drift.

### Seed-based functional connectivity analysis

**Fig. 1:**
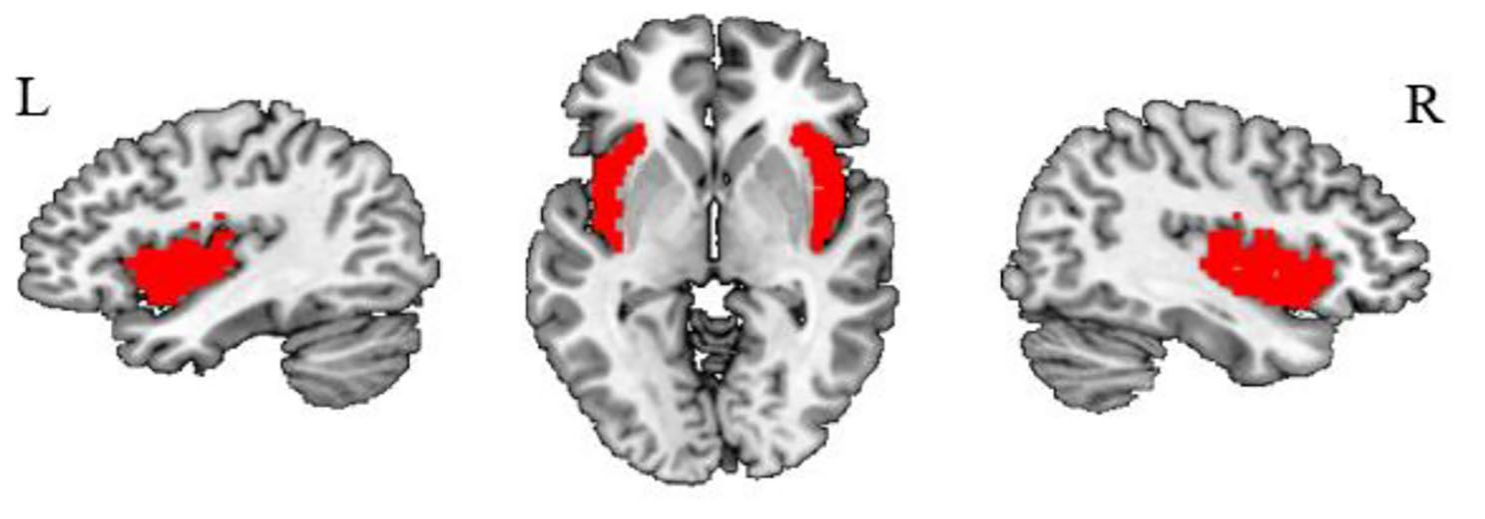
The seed region of the insula per hemisphere in the Anatomical Automatic Labeling (AAL) atlas

To explore the abnormal resting-state functional connectivity of the insula to the voxels of the whole brain between the OCD and HC groups. We selected the bilateral insula as the seed regions of interest (ROIs) using the AAL atlas (Fig. 1).

Seed-based resting-state functional connectivity analysis was performed using the Resting-State fMRI Data Analysis Toolkit software package (RESTplus, version 1.24, http://resting-fmri.sourceforge.net. sourceforge.net). First, the regional time series averaged across all voxels within each seed were extracted. Then, Pearson’s correlation analysis of the time series was performed between the seed reference and the whole brain in a voxel-wise manner to obtain the intrinsic functional connectivity maps of each seed region for all participants. Finally, the subject-level correlation maps were z scored using Fisher r-to-z transformation before taking the average across subjects for further group-level analysis.

### Statistical analysis

#### Group comparisons

General linear model (GLM) was carried out to identify the distinct resting-state functional connectivity patterns of insula between OCD and HC groups, with the age, sex, and head motion as the covariates in SPM12 (https://www.fil.ion.ucl.ac.uk/spm/). The significance threshold was set as *P* < 0.005 (uncorrected) at the voxel level and a familywise error (FWE) corrected *P* < 0.025 (0.05/2) at the cluster level [35, 36], since between-group comparisons of functional connectivity maps were separately performed with two seed ROIs on the left and right insula. Furthermore, to explore the possible effects of previous drug exposure on the functional connectivity of the insula, we excluded 14 patients who had a history of previous medication and performed a secondary group comparison between medication-naïve OCD patients and the HC group using the same method. In addition, statistical analysis of sociodemographic and clinical data was performed using SPSS 24 (SPSS, Inc., Chicago, IL). A two-sample t test was used for continuous variables, and the chi-square test was used for categorical variables when comparing the group differences in sociodemographic data between the two groups (*P* < 0.05).

#### Correlation analysis

The exploratory correlation analysis of the intrinsic functional connectivity strength extracted from the regions showing significant group differences with the duration of illness, age of onset, symptom severity measured by Y-BOCS (including obsession and compulsion subscale), as well as HAMD and HAMA scores in the OCD group were performed to determine whether the insular rsFC abnormalities were correlated with the clinical characteristics. After the linear correlation conditional tests, including the Kolmogorov‒Smirnova normal distribution test, for variables that met the normal distribution and linear condition, we used the Pearson correlation, and those that did not meet the conditions we used the Spearman correlation. An FDR q value < 0.05 was considered statistically significant for multiple comparisons in this correlation analysis. (*P* < 0.05, corrected with FDR).

## Results

The sociodemographic and clinical characteristics of the OCD and HC groups are provided in Table 1. There were no significant differences in age (t = 0.670, *P* = 0.504), sex (χ^2^=-0.156, *P* = 0.876) or head motion (t=-1.407, *P* = 0.161) between the two groups.

**Table 1:** Demographic Characteristics

Characteristic	Group, mean ± SD		Statistics	
	OCD (N = 85)	HC (N = 85)	t/χ^2^	P value
Age (years)	29.18 ± 8.71	28.16 ± 10.85	0.670	0.504
Sex (M/F)	52/33	51/34	-0.156	0.876
Head motion (mm)	0.047 ± 0.028	0.052 ± 0.021	-1.407	0.161
Age of onset (years)	22.08 ± 7.18	-	-	-
Illness of duration (years)	7.09 ± 5.38	-	-	-
Y-BOCS-Total score	21.54 ± 5.47	-	-	-
Y-BOCS-Obsession score	13.06 ± 5.27	-	-	-
Y-BOCS-Compulsion score	8.48 ± 5.34	-	-	-
HAMD-17	9.32 ± 4.76	-	-	-
HAMA-14	9.32 ± 5.32	-	-	-

*Abbreviations*: OCD: obsessive-compulsive disorder; HC: healthy control; SD: standard deviation; HAMD-17: 17-item Hamilton Depression Rating Scale; HAMA-14: 14-item Hamilton Anxiety Rating Scale; Y-BOCS: Yale-Brown Obsessive Compulsive Scale

**Fig. 2:**
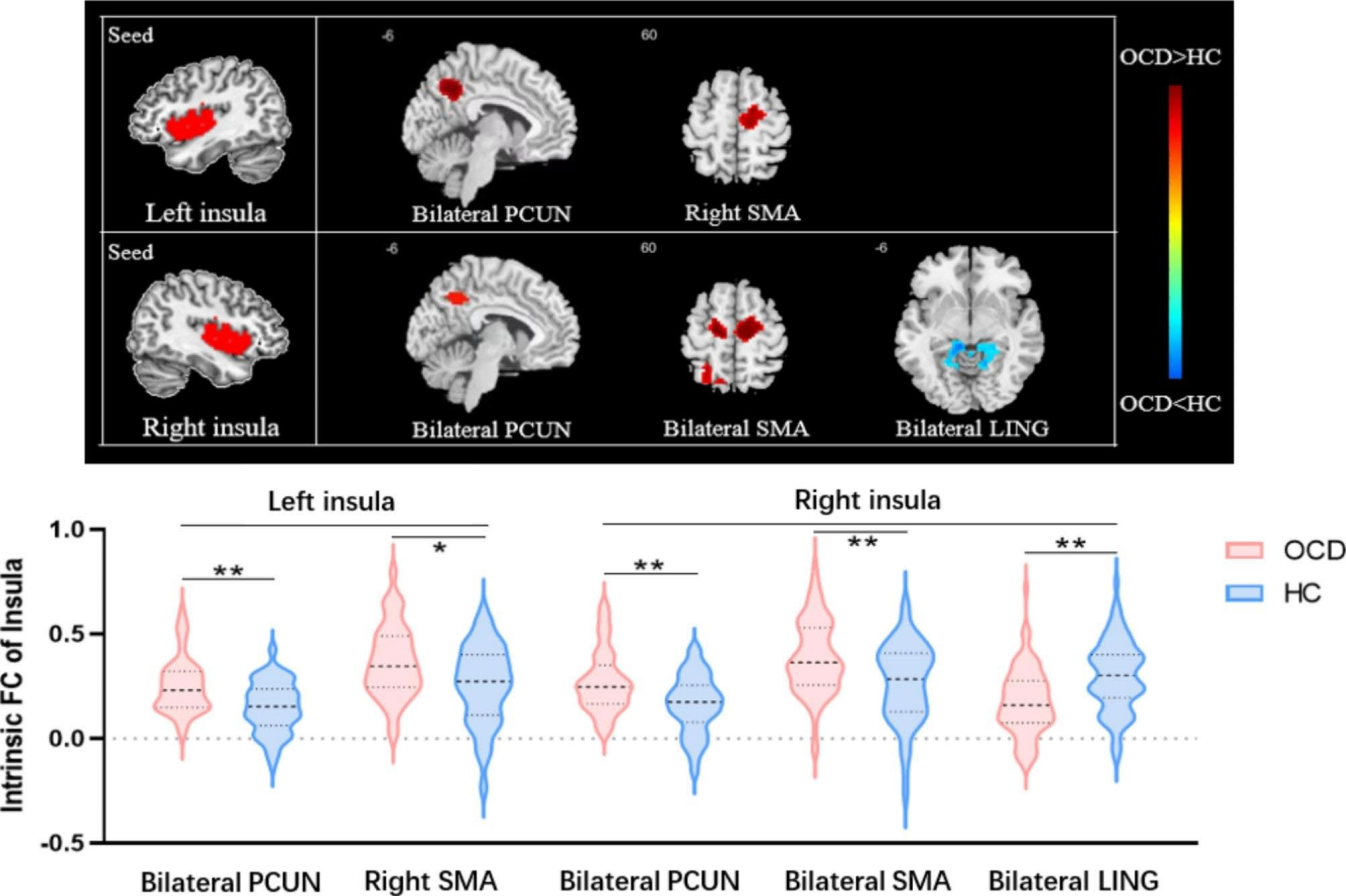
(A). Significantly group-specific regions in intrinsic FC with the insula between the OCD and HC groups. Regions with increased intrinsic FC are shown in red, and those with decreased intrinsic FC are shown in blue. (B) The violin plot represents the intrinsic FC of the insula with each significant region in the OCD and HC groups. (*P_FWE−corrected_ < 0.05, ** *P*_FWE−corrected_ < 0.01) (*Abbreviations*: PCUN: precuneus; SMA: supplementary motor area; LING: lingual gyrus; rsFC: resting-state functional connectivity; OCD: obsessive-compulsive disorder; HC: healthy control; FWE: family-wise error.)

The results of two-sample t test analyses comparing the seed-based resting-state functional connectivity of insula between OCD and HC groups and the exploratory correlation analysis with clinical characteristics were as follows (Fig. 2; Table 2).

**Table 2:** Two-sample t-test results of the whole-brain intrinsic functional connectivity of bilateral insula between the OCD and HC groups

Seed Region	Area	BA	L/R	Voxels	MNI			T peak-values	*P*_uncorrected_	*P*_FWE−corrected_
					X	Y	Z			
Left insula										
OCD > HC	Precuneus/Inferior Parietal Lobule	31/7	L&R	982	-15	-54	42	5.83	0.000	0.000*
	Supplementary motor area	6	R	242	15	-6	57	3.84	0.001	0.019*
Right insula										
OCD > HC	Precuneus/Inferior Parietal Lobule	31/7	L&R	970	-15	-54	42	5.35	0.000	0.000*
	Supplementary motor area	6	L&R	439	-15	-15	54	5.45	0.000	0.000*
OCD < HC	Lingual Gyrus	30/19	L&R	346	36	-42	3	4.04	0.000	0.002*

*Abbreviations*: OCD: obsessive-compulsive disorder; HC: healthy control; BA: Brodmann areas; MNI: Montreal Neurological Institute; R: right; L: left; FWE: familywise error correction

* *P* < 0.025 with FWE correction.

### Group comparison

#### Left insular intrinsic functional connectivity

Compared to the HC group, patients with OCD showed significantly increased positive intrinsic functional connectivity of the left insula and a large cluster including the bilateral precuneus gyrus extending to the inferior parietal lobule. Additionally, we found another significantly enhanced positive intrinsic functional connectivity of the left insula and right supplementary motor area in OCD patients relative to HCs.

#### Right insular intrinsic functional connectivity

In patients with OCD, we observed a significant increase in the positive resting-state functional connectivity between the right insula and the bilateral precuneus/inferior parietal lobule and supplementary motor area. Furthermore, the OCD patients displayed significantly reduced positive intrinsic functional connectivity of the right insula and bilateral lingual gyrus compared to HCs.

#### Secondary analysis of medication-naïve OCD patients

After excluding 14 OCD patients with a history of previous medication, the results of the group comparisons between medication-naïve OCD patients (N = 71) and HC subjects (N = 85) were similar to the primary results from the whole OCD patient group (Supplementary Figure S1).

### Correlation analysis

**Fig. 3:**
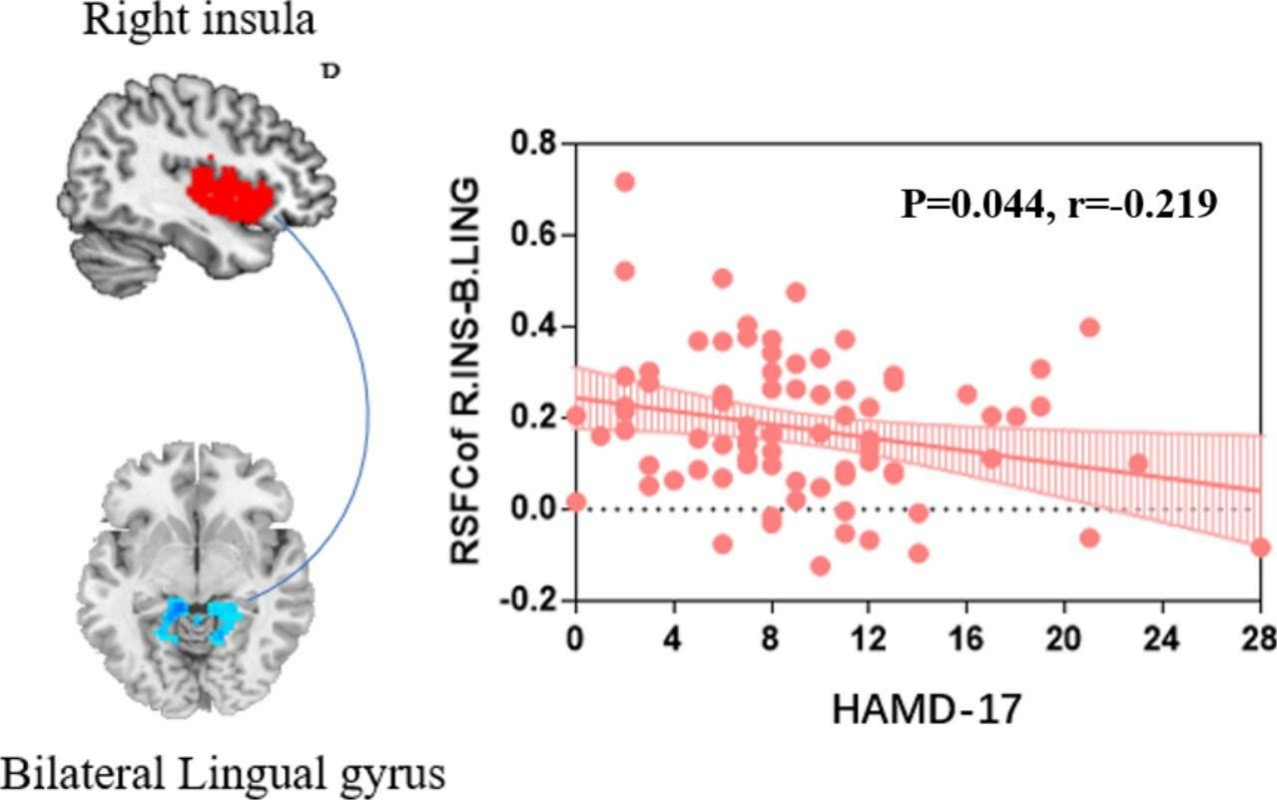
The correlation analysis results of the mean FC values between the right insula and bilateral lingual gyrus and clinical variables in the OCD group. Higher scores of the HAMD-17 (P = 0.044, r=-0.219) were correlated with lower intrinsic rsFC of right insula and bilateral lingual gyrus. The shaded area surrounding the line represents the 95% confidence interval. (*Abbreviations*: OCD: obsessive-compulsive disorder; HAMD-17: 17-item Hamilton Depression Rating Scale; rsFC: resting-state functional connectivity.)

Via correlation analysis, we revealed that OCD patients demonstrated a trend of correlation between the decreased intrinsic functional connectivity of the right insula and bilateral lingual gyrus and the increased HAMD scores (r=-0.219, *P* = 0.044, uncorrected) (Fig. 3).

## Discussion

In the current study, using the seed-based resting-state functional connectivity method, we revealed distinct patterns of intrinsic insular functional connectivity alterations in OCD. There were two main findings in this study. First, compared to the HC group, patients with OCD showed significantly increased intrinsic functional connectivity between the bilateral insula with a large cluster including the bilateral precuneus gyrus extending to the inferior parietal lobule and another cluster supplementary motor area. Significantly decreased intrinsic functional connectivity was found only between the right insula and bilateral lingual gyrus in OCD patients relative to HC, which was negatively correlated with HAMD scores rather than YBCOS scores in the OCD group. Our findings indicated the involvement of aberrant insular intrinsic connectivity in OCD patients, and more importantly, the dysconnectivity of insula may be related to the depressive symptoms rather than the obsession or compulsory manifestation in OCD.

### Hyperconnectivity of the bilateral insula and precuneus

Relative to the HC group, the OCD patients showed increased intrinsic functional connectivity between the bilateral insula and bilateral precuneus extending to the inferior parietal lobule. The precuneus and inferior parietal lobule are both important core regions of the default mode network for integrating and processing self-referential information [37, 38]. Because the symptoms of OCD are triggered mostly by internal intrusive thoughts or images rather than external stimuli [39], OCD patients have been reported to have difficulties with the deactivation of the DMN at rest [40]. In addition, a previous study of static spontaneous brain activity also revealed increased amplitudes of low frequency fluctuation (ALFF) in the insula and precuneus [41]. Greater intrinsic connectivity between the insula and the precuneus or inferior parietal lobule in OCD patients has been reported in several fMRI studies [25, 42, 43], which was consistent with our results.

### Hyperconnectivity of the bilateral insula and supplementary motor area

Patients with OCD demonstrated enhanced intrinsic connectivity of the bilateral insula and supplementary motor area compared with HC subjects. The supplementary motor area, which may be associated with the deficient inhibitory control of compulsory or obsessive behavior, has been found to be hyperactive in OCD patients according to prior studies [44, 45]. In a previous structural study, researchers found a thicker cortex in the presupplementary motor area and right anterior insula in OCD patients [46]. In addition, several prior task-fMRI studies demonstrated that OCD patients exhibited longer inhibitory control reactive time and more inhibitory control errors than healthy controls and overactivation in the supplementary motor area, presupplementary motor area and anterior insula/frontal operculum during error processing, as well as deactivation of the anterior insula/frontal operculum and supplementary motor area regions during inhibitory control tasks, which may be an underlying neuroimaging mechanism of impaired inhibitory control performance in OCD [47, 48]. Combined with the previous findings, our result of higher intrinsic functional connectivity of the insula and SMA might be speculatively associated with the deficits of inhibitory control to OCD-related behaviors. However, one recent study performed by the OCD consortium including large samples of patients reported a hypoconnectivity between the insula and the SMA [49]. This inconsistency to our findings may be due to the differences of sample characteristics. The OCD consortium performed mega-analysis by collecting independent samples from different research institutes, and the included patients were varied in clinical characteristic including age range, medication and various severity of symptoms. In contrast, the participants in our study were the comorbidity-free and unmedicated adult patients with relatively mild OCD symptoms (YBOCS = 21.54 < = 25), thus the results of our current may represent the functional brain mechanism underlying the pathophysiology of such OCD patients.

### Hypoconnectivity of the right insula and lingual gyrus

Significantly decreased intrinsic connectivity of the right insula and bilateral lingual gyrus was observed in OCD patients compared to HCs. The lingual gyrus is located in the visual cortex region and may contribute to emotional perception during visual stimulation and the further processing of complex visual information [50, 51]. In addition to involving deficits in cognitive and behavioral inhibitory control, OCD is also recognized to be associated with visual processing impairments [52], which may be related to abnormalities in the lingual gyrus. Alterations in the brain structures of the lingual gyrus have been reported in several studies, such as a thinner cortex and a smaller surface area of the lingual gyrus [53, 54]. Another morphological meta-analysis revealed that the gray matter volume changes in the lingual gyrus and motor regions were more specific to OCD than to schizophrenia- and autism-spectrum disorders [55]. In previous rs-fMRI studies, researchers observed a lower centrality degree of the lingual gyrus [56, 57], and the ALFF values were found to be increased in the insular cortex but decreased in the lingual gyrus of OCD patients, which was associated with symptom severity [41, 58]. These abnormal local activity patterns could be normalized by coping through cognitive therapy [59].

Interestingly, we further observed that there was a trend of correlation between the reduced intrinsic connectivity of the right insula and lingual gyrus and the enhanced severity of depressive symptoms rather than OCD-related symptoms in the OCD group. Previous studies have reported reduced insular volume in OCD patients with comorbid depression [60], and the activity of the lingual gyrus could distinguish OCD patients from controls during multi-emotion analysis [61]. This finding suggested that we should not neglect the role of the insula and lingual gyrus in the depressive symptoms of OCD in future studies. However, the association of right insula-lingual gyrus connectivity and depressive symptoms in OCD group did not survive multiple comparison correction and should be interpreted with caution.

## Limitations

The current study has several features that merit consideration in the interpretation of the results. First, our study had a cross-sectional design and was unable to investigate the long-term changes in insular connectivity patterns and the effects before and after treatments in OCD patients. In the future, longitudinal studies need to be performed to explore the functional connectivity of the same ROIs at baseline and after first-line treatments for OCD and verify the engagement of these regions in pharmacological and behavioral treatments. Second, our current results were derived from a single study, and future studies in unmedicated OCD samples with no comorbidities or comorbidities limited to depressive disorders are needed to replicate and validate our findings. Third, the correlation between the altered intrinsic connectivity of the insula and clinical features should be treated with caution, as they did not survive FDR correction for multiple comparisons. Note that these results still provide valuable insights that there is a trend for depressive symptoms in OCD to be associated with dysconnectivity of the insula, which could guide future research. Finally, this study used the entire insula region as the seed. However, several studies have implicated the distinct functions of different insular subregions [62]. In future studies, the intrinsic connectivity patterns of finer-grained insular subfields should be further explored in OCD to extend our study.

## Conclusion

Using the seed-based resting-state connectivity approach, our present research identified alterations in the intrinsic connectivity patterns of the insula in OCD patients compared to healthy controls. We found that increased insular intrinsic functional connectivity mainly located in the bilateral precuneus gyrus and supplementary motor area, and decreased intrinsic connectivity only between the right insula and bilateral lingual gyrus, which was associated with the severity of depressive symptoms in OCD patients. Our findings provide the neuroimaging evidence for a pathophysiological role of the insula in OCD and suggest that future studies focusing on depressive manifestation in patients with OCD should consider the role of the insula in this regard.

## Electronic supplementary material

Below is the link to the electronic supplementary material.

Supplementary Material 1: Supplementary Figure S1

## Data Availability

The datasets used and analyzed during the current study are available from the corresponding author on a reasonable request.
