# Supplementary material for: Abnormal resting-state functional connectivity of the insula in medication-free patients with obsessive-compulsive disorder
Source: BMC Psychiatry. 2022 Nov 29;22:742. doi: 10.1186/s12888-022-04341-z (PMC9710058; doi:10.1186/s12888-022-04341-z)
Supplement: Supplementary file 1 — Supplementary Material 1: Supplementary Figure S1 [file 12888_2022_4341_MOESM1_ESM.docx]

**Supplementary Figure S1**


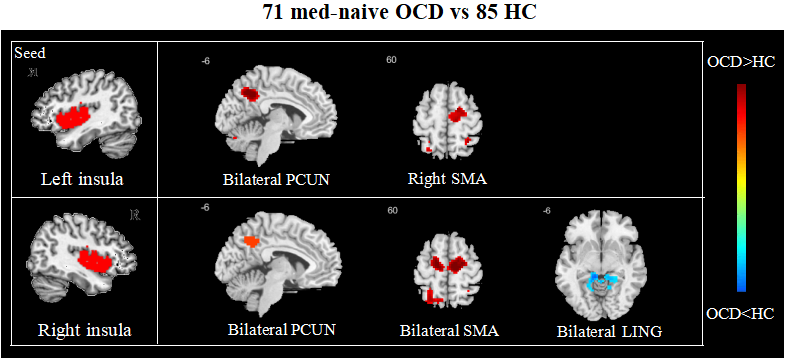


**Figure S1** The results of the group comparisons between medication-naive OCD patients (N=71) and HC subjects (N=85). Regions with increased intrinsic FC are shown in red, and those with decreased intrinsic FC are shown in blue. (*P*_FWE-corrected_ < 0.025).

*Abbreviations*: rsFC: resting-state functional connectivity; SMA: supplementary motor area; LING: lingual gyrus; PCUN: precuneus; rsFC: resting-state functional connectivity; OCD: obsessive-compulsive disorder; HC: healthy control; FWE: family‐wise error.
